# Customizable, engineered substrates for rapid screening of cellular cues

**DOI:** 10.1088/1758-5090/ab5d3f

**Published:** 2020-02-07

**Authors:** Eline Huethorst, Marie FA Cutiongco, Fraser A Campbell, Anwer Saeed, Rachel Love, Paul M Reynolds, Matthew J Dalby, Nikolaj Gadegaard

**Affiliations:** 1 Division of Biomedical Engineering, School of Engineering, University of Glasgow, Glasgow, G12 8LT, United Kingdom; 2 Institute of Cardiovascular and Medical Sciences, University of Glasgow, Glasgow, G12 8QQ, United Kingdom; 3 Division of Electronics and Nanoscale Engineering, School of Engineering, University of Glasgow, Glasgow, G12 8LT, United Kingdom; 4 Centre for the Cellular Microenvironment, University of Glasgow, Glasgow, G12 8QQ, United Kingdom; Nikolaj.gadegaard@glasgow.ac.uk

**Keywords:** nanofabrication, injection moulding, cell morphology, substrate rigidity, topography, high throughput, pillar

## Abstract

Biophysical cues robustly direct cell responses and are thus important tools for *in vitro* and translational biomedical applications. High throughput platforms exploring substrates with varying physical properties are therefore valuable. However, currently existing platforms are limited in throughput, the biomaterials used, the capability to segregate between different cues and the assessment of dynamic responses. Here we present a multiwell array (3 × 8) made of a substrate engineered to present topography or rigidity cues welded to a bottomless plate with a 96-well format. Both the patterns on the engineered substrate and the well plate format can be easily customized, permitting systematic and efficient screening of biophysical cues. To demonstrate the broad range of possible biophysical cues examinable, we designed and tested three multiwell arrays to influence cardiomyocyte, chondrocyte and osteoblast function. Using the multiwell array, we were able to measure different cell functionalities using analytical modalities such as live microscopy, qPCR and immunofluorescence. We observed that grooves (5 *μ*m in size) induced less variation in contractile function of cardiomyocytes. Compared to unpatterned plastic, nanopillars with 127 nm height, 100 nm diameter and 300 nm pitch enhanced matrix deposition, chondrogenic gene expression and chondrogenic maintenance. High aspect ratio pillars with an elastic shear modulus of 16 kPa mimicking the matrix found in early stages of bone development improved osteogenic gene expression compared to stiff plastic. We envisage that our bespoke multiwell array will accelerate the discovery of relevant biophysical cues through improved throughput and variety.

**Nomenclature**
ACAN: aggrecan
APD50: action potential duration at 50% of the amplitude
CD50: contraction duration at 50% of the amplitude
CHF_3_/Ar: trifluoromethane/argon
COL1A: collagen type 1a
COL2A: collagen type 2a
CoV: coefficient of variation
DMEM: Dulbecco’smodified Eagle’s medium
EBL: electron beam lithography
fps: frames per second
IPA: isopropyl alcohol
MIBK: methyl isobutyl ketone
NiCr: nichrome
NIL: nanoimprint lithography
NMP: N-Methyl-2-pyrrolidone
OCN: osteocalcin
OPN: osteopontin
qPCR: quantitative polymerase chain reaction
RIE: reactive ion etching
RUNX2: Runx family transcription factor 2
SD: standard deviation
SEM: scanning electron microscope
SF6/C4F8: sulfur hexafluoride/octafluorocyclobutane
SOX9: SRY-box 9
*T*_Contraction_: contraction time
*T*_Relaxation_: relaxation time

## Introduction

Through its ability to regulate cell behavior, the cellular micro-environment plays a key role in health and disease [1–4]. Manipulation of the cell micro-environment using biochemical and biophysical cues is therefore widely explored as a means to alter cell behavior both *in vitro* and *in vivo* [5–8]. Of particular interest are engineered substrates precisely and reproducibly made with defined biophysical properties [9–11]. Substrates that recapitulate substrate rigidity or surface topographical cues present in the cell environment have been shown *in vitro* to force cells to behave differently [12–14]. Yet even interaction of cells with artificial biophysical environments (i.e. topography or substrate rigidity not found in the natural cell niche) can powerfully change cell behavior by inducing cell signaling mechanisms through mechanotransduction [15–18]. Artificial biophysical environments have therefore been shown to preferentially direct mesenchymal stem cell differentiation [19–21], alter endothelial cell functionality [22–24] and change in neurogenic subtype [14, 25].

Discovery of biologically-relevant engineered substrates has long relied on the use of individual substrates assessed in tandem to screen for positive hits but is severely hindered in throughput. In recent years, combinatorial libraries of biomaterials, including topographies, have been made to increase efficiency of screening [26–29]. However, these high-content platforms lack physical segregation between or isolation of substrates of interest. Continuous exchange of signaling molecules between cells on different engineered substrates makes it impossible to uncouple biophysical and paracrine based effects. In addition, these combinatorial libraries have bespoke dimensions incompatible with most analytical laboratory equipment. New platforms that allow rapid and high-throughput screening of a library of materials are thus required. A good screening platform should also be able to isolate the effect of a specific biophysical cue to limit confounding paracrine effects in response to other biophysical cues [30, 31] and should be made from a biocompatible material. Moreover, these screening platforms need to be highly generalizable across substrates, cell types and various regenerative medicine applications. The screening platform should additionally allow a wide variety of validation assays for thorough selection of the most appropriate features for possible translational application.

In this study, we present a new platform for rapid screening of a wide variety of biophysical cues. The multiwell array is a robust and high throughput platform based on thermoplastics such as polystyrene, with the footprint and dimensions of a 96-well plate. The complete multiwell array is a fully customizable slide welded to a bottomless well plate, both of which were manufactured through injection moulding. This allows for an industrial level production of biocompatible substrates with low cost and high reproducibility. The multiwell array is presented in a 96-well format, allowing various biological assays to be carried out with standard laboratory tools and techniques. This includes quantitative polymerase chain reaction (qPCR), fluorescent immunochemistry and microscopy. With this design, 24 different topographies or rigidities, each one isolated in a well, can be simultaneously compared without confounding from paracrine signals between samples.

To demonstrate the broad range of possibilities that the platform offers, we assessed three distinct substrates to alter the behavior of three different cell types. We created multiwell arrays that vary the type of biophysical cue (topography versus substrate rigidity), anisotropy or geometry (gratings versus pillars) and length scale (nanometer versus micrometer) presented to the cells. We cultured human induced pluripotent stem cell-derived cardiomyocytes (hiPSC-CMs) on nano- and micro-grooves, as groove structures was shown to maturate cardiomyocytes [32, 33]. The adhesion of chondrocytes influences chondrogenic viability and quality, and could therefore be influenced by reducing cell area, confinement and adhesion [32, 33]. We finally tested a variety of substrate rigidities (varied using high aspect ratio pillars) [34, 35] on osteogenic differentiation, a process shown to be tightly controlled by the stiffness of the microenvironment [36, 37]. Thus, the multiwell array presents an alternative screening platform for rapid, accurate and highly reproducible interrogation of new engineered microenvironments.

## Results

### Customization of the multiwell array

**Figure 1.**
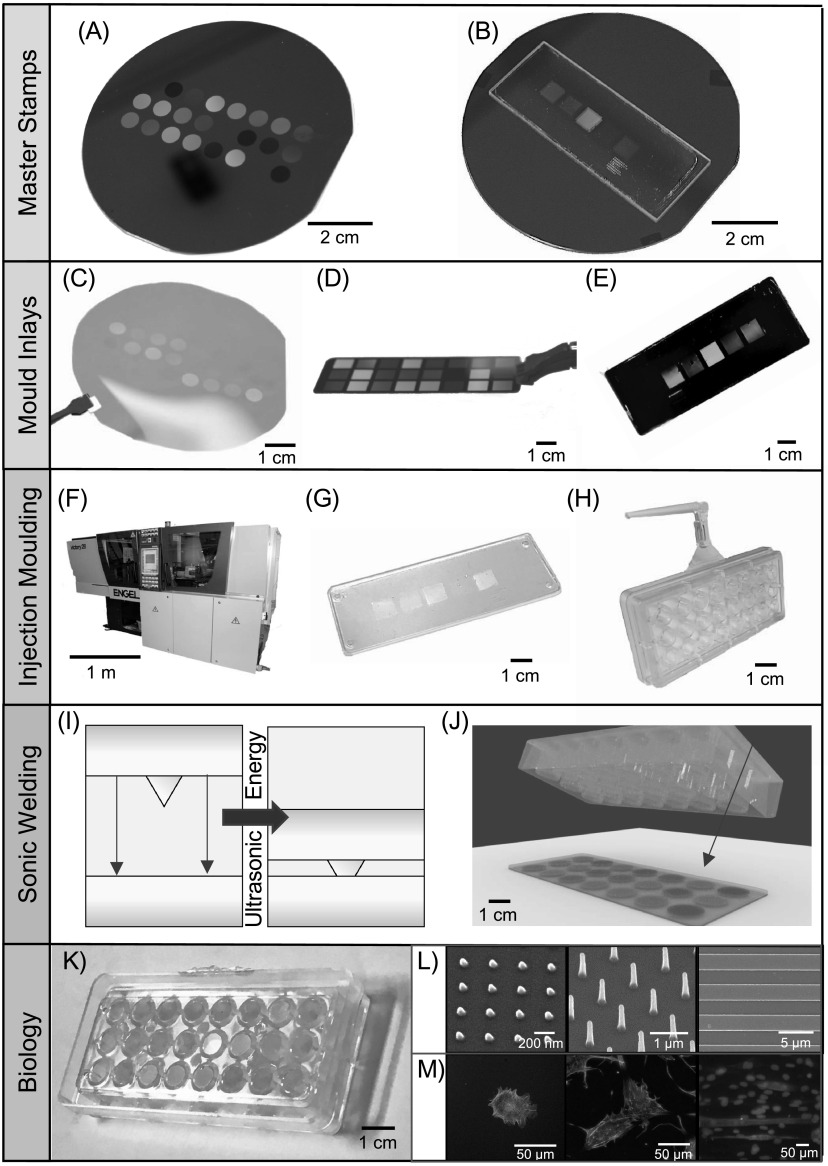
Bespoke multiwell array fabrication for rapid screening of rigidity or topographical cues. We illustrate the various stages involved in the fabrication of our highly customizable multiwell array. See Experimental section for the detailed processes. First, master stamps are fabricated with the desired patterns on (A) a silicon wafer or (B) a quartz slide. The initial pattern is formed using electron beam lithography (EBL) and metal lift-off, and then etched into features using a plasma etching process. Pillars, pits or grooves can be defined on master stamps to fabricate multiwell arrays that provide topographical cues. High aspect ratio pillars can be defined on master stamps to provide controlled changes in substrate rigidity. Afterwards, a negative relief of the master stamp is fabricated by (C) nanoimprint lithography (NIL) to make a SmartNIL (EVG) foil, (D) electroplating of nickel or (E) NIL of SU-8 epoxy photoresist on Cirlex^®^ polyimide [38]. The resulting nickel or polymer replica are used in an (F) industrial grade injection moulding Engel Victory tool, which (G) moulds thermoplastic polymers such as polystyrene or polycarbonate to replicate structures of the original master stamp onto a slide. In this paper, we focus on the use of polymeric replicates as moulds for injection moulding to prevent any adverse cell effects from nickel. (H) A bottomless well plate with 8 columns × 3 row and approximately 0.3 cm^2^ growth area (similar to standard 96 well plate) was also made from injection moulding of polystyrene. (I), (J) To unite the slide and the bottomless well plate, the two are brought into contact and ultrasonic energy is used to melt a weld seam on the plate into the replica to form a joint around the patterned area. (K) The multiwell array combines multiple types of biophysical cues in one plate, (L) e.g. nano-pillars, tall pillars or nano- and micro-grooves, and (M) multiple cell types. This allows for high-throughput screening of isolated cues without risk of paracrine signalling between samples confounding the effects of topography or rigidity. The standard well plate format of the multiwell array allows established analytical techniques such as microscopy to be performed easily.

The multiwell array is comprised of two parts, each fully customizable in design. An overview of the fabrication process is depicted in figure 1 and detailed in the Experimental section. First, topographies or rigidities of interest (defined by patterns) are created on a slide through a multistep engineering process. A master stamp containing the patterns of interest are defined on silicon (figure 1(A)) or quartz (figure 1(B)) through standard fabrication techniques of electron beam lithography (EBL) and plasma etching. The master stamps are customized by combining different shapes (i.e. pits, pillars and grooves) and length scales from nano- to micrometer sizes depending on user specifications.

To enable high-throughput production of the engineered substrate, the master stamps are used to create negative relief replica, which is thereafter utilized as a mould inlay for injection moulding (figures 1(C)–(E)). The mould inlay is normally prepared from a polymeric material to withstand high temperatures and high pressures required for high fidelity replication using injection moulding (figure 1(F)) [35]. In this paper, we focused on using polymeric mould inlays for injection moulding to preclude cytotoxicity from nickel. From one mould inlay, hundreds of slides containing the patterns as the original master stamp per hour are made through injection moulding (figure 1(G)). We have not seen deterioration of the mould inlay after hundreds to thousands of replicates. But when needed, a new master stamp can be fabricated or a master stamp can be used again to create a new mould inlay for further production of slides.

Aside from the engineered substrate slide containing patterns, the well plate format can be easily tuned to match the scale of the experiment required by the end user. The bottomless well plate is also produced through the same high throughput injection moulding process. The dimensions and arrangement of the patterns on the master stamp are set to match the specifications of the desired well plate format. In this study, we focus on creating a multiwell array, containing 24 wells with a 96-well format (0.3 cm^2^ per well), which is one of the most commonly used and preferred formats for automated and high throughput screening (figure 1(H)) [39].

For a fully enclosed device, the two components are joined together through ultrasonic welding (figures 1(I) and (J)). Since the slide and bottomless well plate are made separately, one can mix and match different combinations of the two components easily. Here, we created multiwell arrays that presented different nanopillars, grooves or high aspect ratio pillars all in the same 96-well format. These topographies and rigidity cues incorporated in the multiwell array were then used to test changes in functionality of different cell types. To show the utility of our customized multiwell array we developed polystyrene and polycarbonate slides patterned with varying topographies and rigidities and screened them on the behavior of cardiomyocytes, chondrocytes and osteoblasts.

### Multiwell array for physiological real-time assessment of hiPSC-CMs function

**Figure 2.**
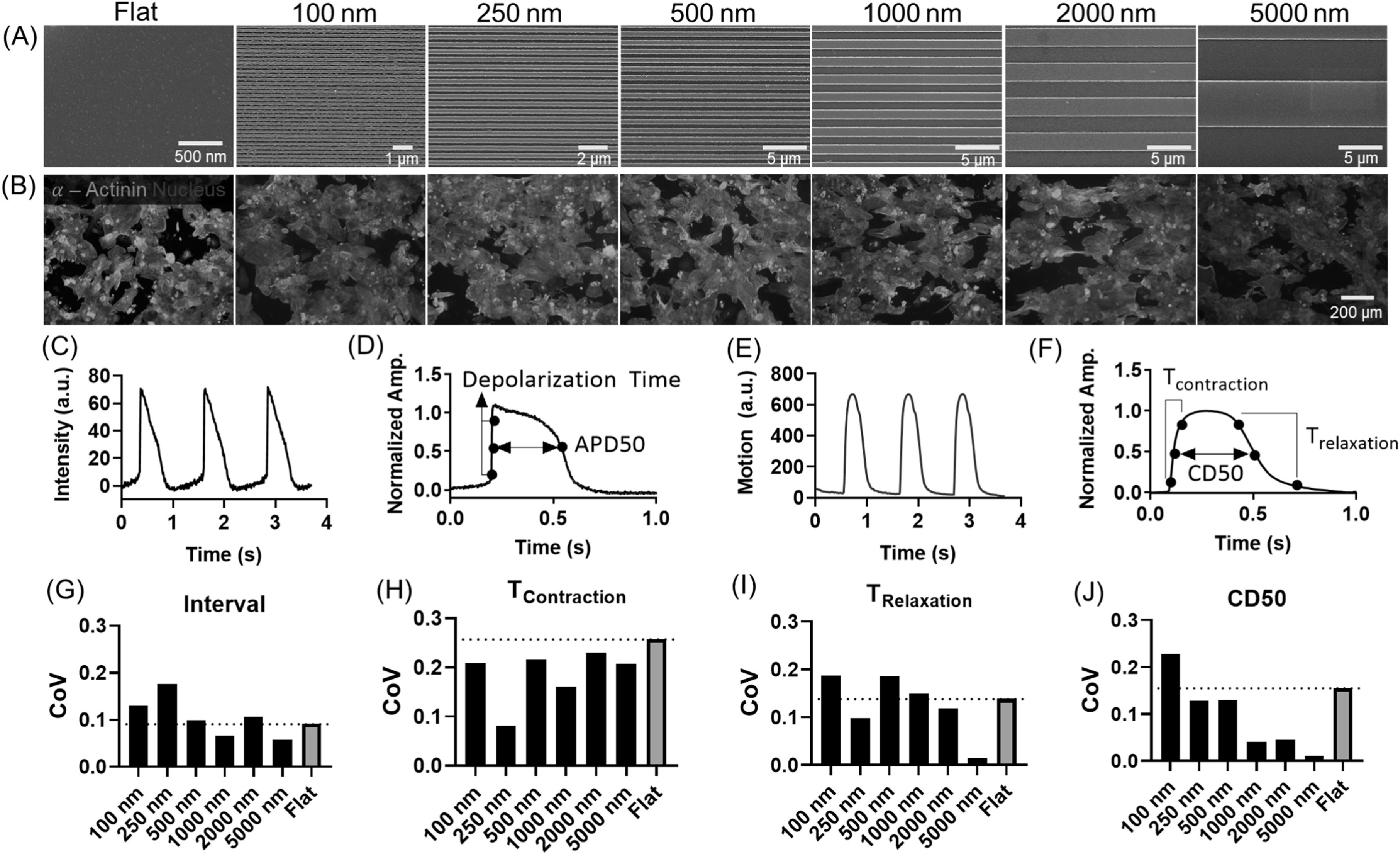
Groove topography reduces the variability in contractile behavior of human induced pluripotent stem cell derived cardiomyocytes (hiPSC-CMs). hiPSC-CMs were grown on the multiwell groove array for 10 d before immunofluorescent staining and functional assessment using high speed microscopy. (A) Scanning electron micrographs (SEM) of grooves with widths of 100, 250, 500, 1000, 2000 or 5000 nm. All grooves had constant depth of 250 nm and width:pitch ratio at 1:1. (B) Fluorescent images of hiPSC-CMs stained for *α*-actinin (green) and DAPI (blue) on corresponding groove topographies. (C)–(F) Functionality of hiPSC-CMs was assessed by measuring voltage (C) and (D) and contractile function (E) and (F). (C) Example trace of 3 action potentials measured over time. (D) Graph explaining electrophysiology parameters Depolarization time and Action Potential Duration (ADP50). (E) Example trace of 3 contractions measured over time. (F) Graph explaining contractility parameters contraction time (*T*_Contraction_), relaxation time (*T*_Relaxation_) and contraction duration at 50% of the amplitude (CD50). (G)–(J) Coefficient of variation (CoV) calculated from standard deviation divided by the mean. CoV was calculated from measurement of interval (G), *T*_Contraction_ (H), *T*_Relaxation_ (I) and CD50 (J), all of which describe contractile behavior.

HiPSC-CMs have been shown to elongate when cultured on microgrooves [40]. Various reports have shown this morphological change to improve functionality towards a more mature phenotype [41–43]. As hiPSC-CMs exhibit a relatively immature phenotype compared to adult CMs, this strategy could be used to induce functional maturation of hiPSC-CMs. In our previous study we used a gradient of grooves and showed that a range of dimensions (8–30 *μ*m wide) improved hiPSC-CMs elongation [40]. However, it is possible that these results could have been influenced by cross-talk of paracrine factors from different hiPSC-CMs functionalities. Here, we used a multiwell array with each groove topography isolated in a well to understand how groove size influences cardiomyocyte phenotype. From each well in the multiwell groove array, we measured hiPSC-CMs morphology and functionality using live microscopy. Because hiPSC-CMs previously increased maturity on the most narrow features (8–30 *μ*m wide) [1], we chose to use a multiwell array with similar and narrower groove widths of 100, 250, 500, 1000, 2000 and 5000 nm and a width:pitch ratio of 1:1 (figure 2(A)). The groove depth was kept constant at 250 nm. We used a Flat surface as a control.

HiPSC-CMs functionality was measured in terms of contractility and electrophysiology. Example traces over time for intensity of voltage-sensitive dyes is shown in figure 2(C) and explained in figure 2(D). An example trace of contractility over time is given in figure 2(E) and explained in figure 2(F). Contractility of hiPSC-CMs was visibly influenced by different groove dimensions, as shown in the supplementary videos which are available online at stacks.iop.org/BF/12/025009/mmedia. The nano- and micro-grooves did not significantly affect hiPSC-CMs morphology (figure 2(B)), electrophysiology (supporting table 1) or contractile behavior (supporting table 2) compared to FLAT after 10 d.

HiPSC-CMs and other iPSC derived cell types are known for their variability as a result of differences in donor and the protocols used for dedifferentiation [44–47]. While increasingly endorsed as a physiologically relevant platform for drug screening, the inherent variability in iPSC response is highly undesirable as rigorous drug testing processes require minimal well-to-well variation [48, 49]. Thus, we calculated the coefficient of variation (CoV) across all measures of contractile behavior of hiPSC-CMs on groove topographies by normalising the standard deviation to the mean (figures 2(G)–(J)). For all contractility parameters, CoV measured from hiPSC-CMs on 5000 nm grooves was lowest among all groove topographies compared to Flat (figures 2(G)–(J)). Sum of the CoV across measurements showed that 5000 nm grooves (sum CoV = 0.291) induced the lowest variation among all groove topographies compared to Flat (sum CoV = 0.644). Even though 5000 nm grooves reduced variation most drastically, variability of contractility measurements was also reduced by other groove substrates compared to FLAT. A trend towards reduced variability with increased groove size was also apparent across most contractility measures (figures 2(G), (I) and (J)), and summed values of CoV (100 nm = 0.755, 250 nm = 0.485, 500 nm = 0.632, 1000 nm = 0.417, 2000 nm = 0.5) The reduction in well-to-well variability induced by 5000 nm groove topography may be invaluable as a high quality tool for presenting functional cardiomyocytes for drug screening.

### Improved chondrogenic maintenance using nanopillars

**Figure 3.**
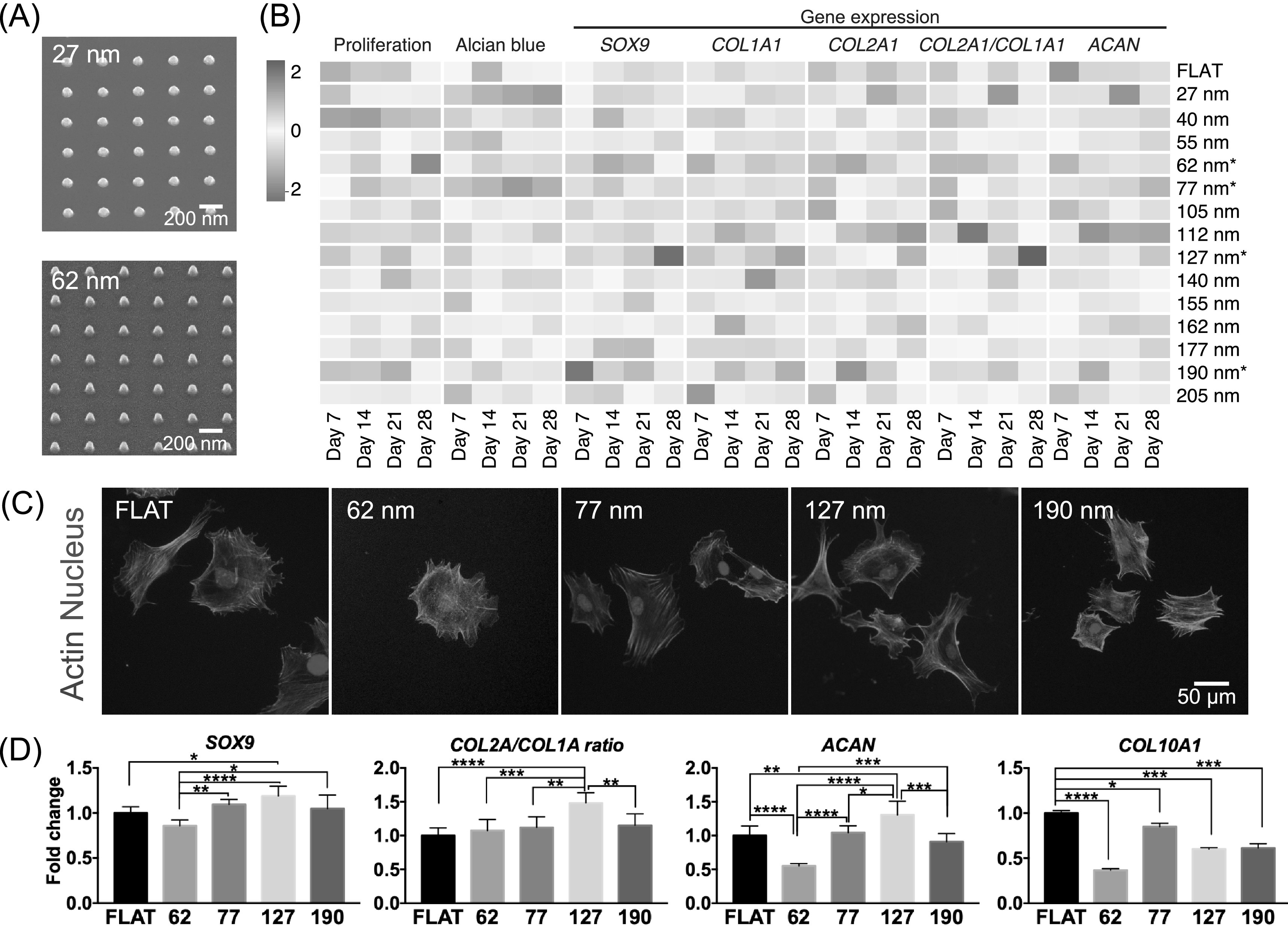
Tall nanopillars aid chondrogenic maintenance. A multiwell array containing nanopillars with constant 300 nm pitch, constant 100 nm diameter and varying heights were used to screen for topographies that improved or maintained chondrogenic properties of primary chondrocytes. Numbers denote the height of nanopillars examined. (A) SEM of representative nanopillars with heights of 27 and 62 nm found on the multiwell array. (B) Multimodal analysis of chondrogenic function induced by nanopillars over 28 d. A heatmap showing time point analysis of proliferation, matrix deposition and gene expression changed by nanopillars of varying height. Cultured chondrocytes were propagated in tissue culture plastic for 7 d before growth on the multiwell array to determine nanopillars that can reverse chondrogenic dedifferentiation. Chondrocyte behavior was measured at day 7 intervals for 28 d. Each tile represents the mean value of each measurement at a given time point across 2 independent experiments (*n* = 4). Nanopillars that significantly changed behavior of cultured chondrocytes were selected for further examination. (C) Representative images of cultured chondrocytes on specific nanopillar heights after 24 h. Cultured chondrocytes were fixed and stained against actin (green) and the nucleus (blue). Quantification of chondrocyte morphology is presented in figure S1. (D) Expression of chondrogenic genes in primary chondrocytes after culture on selected nanopillars for 14 d. Gene expression data on chosen nanopillars are presented as mean ± SD across 3 independent experiments (*n* = 6). A comparison of gene expression between cultured and primary chondrocytes grown on selected nanopillars for 14 d is presented in figure S2. Statistical significance was calculated using one-way ANOVA with Tukey’s post hoc test with * denoting *p* < 0.5, ** denoting *p* < 0.05, *** denoting *p* < 0.005, **** denoting *p* < 0.0001.

Loss of chondrocyte phenotype and dedifferentiation into fibroblasts, commonly observed on standard tissue culture plastic, is exacerbated by increased adhesion to the substrate [32, 33]. We hypothesized that reduction of chondrocyte adhesion using nanopillars improves chondrocyte phenotype. A multiwell array with 14 nanopillar types (with fixed dimensions of 100 nm diameter and 300 nm pitch, and height varying from 27 to 205 nm) were used (figure 3(A)). Using a variety of functional assays, we tested the effect of the nanopillars in reversing dedifferentiation of chondrocytes previously cultured on tissue culture plastic (‘cultured chondrocytes’, figure 3(B)). For visualization and ease of comparison, the data were presented in a heatmap. Over 28 d of culture, we observed that nanopillars with 62, 77, 127 and 190 nm heights changed chondrocyte behavior significantly. Compared to shorter nanopillars, cultured chondrocytes on tall nanopillars (height ≥ 127 nm) generally exhibited decreased proliferation and increased glycosaminoglycan deposition, indicating increased commitment of cells to the chondrocytic lineage [50]. Chondrogenic function was also observed through gene expression analysis, where expression of SRY-Box 9 *(SOX9)* and collagen 2*α*1/collagen 1*α*1 (*COL2A1/COL1A1)* ratio was enhanced on nanopillars with 127 nm height compared to Flat. Expression of aggrecan (*ACAN*), a proteoglycan secreted by mature chondrocytes, and *SOX9* were also significantly upregulated by nanopillars with 190 nm height. On the other hand, chondrogenic genes *SOX9* and *COL2A1/COL1A1* ratio was minimized on 62 and 77 nm tall nanopillars after 28 d indicating fibroblastic phenotype.

Cultured chondrocytes stained against the actin cytoskeleton also revealed changes in cell morphology introduced by varying nanopillar heights (figure 3(C), figure S1). Cultured chondrocytes on 62 and 77 nm heights generally showed statistically similar size to Flat, while cells on 190 nm high nanopillars were larger in size. Actin arrangement into fibers, reflected in actin texture, were higher on chondrocytes grown on 62, 77, 127 and 190 nm tall nanopillars compared to Flat. In contrast to the taller 127 and 190 nm high nanopillars, cells on 62 and 77 nm tall nanopillars showed decreased uniformity in the arrangement of the actin cytoskeleton. This difference indicates anisotropy in actin arrangement and perhaps the generation of intracellular tension as a mechanism that differentiates the effects of shorter and taller nanopillars.

We then further selected nanopillars with 127 and 190 nm heights to improve the maintenance of freshly isolated primary (‘primary’) chondrocytes compared to standard tissue culture plastic (figure 3(D)). We also included nanopillars with 62 and 77 nm heights as controls that were expected to deteriorate chondrogenic maintenance. Using primary chondrocytes we observed similar results as the cultured chondrocytes. At 14 d, *SOX9* and *COL2A1* and *ACAN* expression was significantly upregulated in primary chondrocytes by 127 nm high nanopillars compared with Flat. *COL1A1* expression was significantly reduced in 127 nm high nanopillars compared to Flat. Thus, the collagen *COL2A1*/*COL1A1* ratio in primary chondrocytes was significantly upregulated in 127 high nanopillars. However, all nanopillars reduced *COL10A1* expression in primary chondrocyte. Additionally, we compared the effect of the selected nanopillars on primary and cultured chondrocytes (figure S2) at day 14. We consistently observed upward trends in gene expression from the primary to the cultured chondrocytes grown on 190 nm pillars. Surprisingly, when comparing between the two chondrocyte cell types, we also observed enhancement of *SOX9* and *COL2A1* expression on 62 nm. Similar to other reports [6, 22, 51, 52], our results highlight how cell response to topography is highly dependent on intracellular context, even between cells of the same functional type.

Using the multiwell nanopillar array we systematically screened for an optimal nanopillar for chondrogenic differentiation and maintenance using a wide variety of standard analytical assays. Generally, we observed chondrogenic maintenance of primary isolated chondrocytes improved by nanopillars with 127 nm height compared to Flat. Nanopillars with 127 nm height represents a possible new material that could be used for sustained *in vitro* culture of primary chondrocytes without the need for expensive biochemical cues such as transforming growth factor beta.

### Substrate stiffness mimicked by nanopillars directs osteogenic differentiation

**Figure 4.**
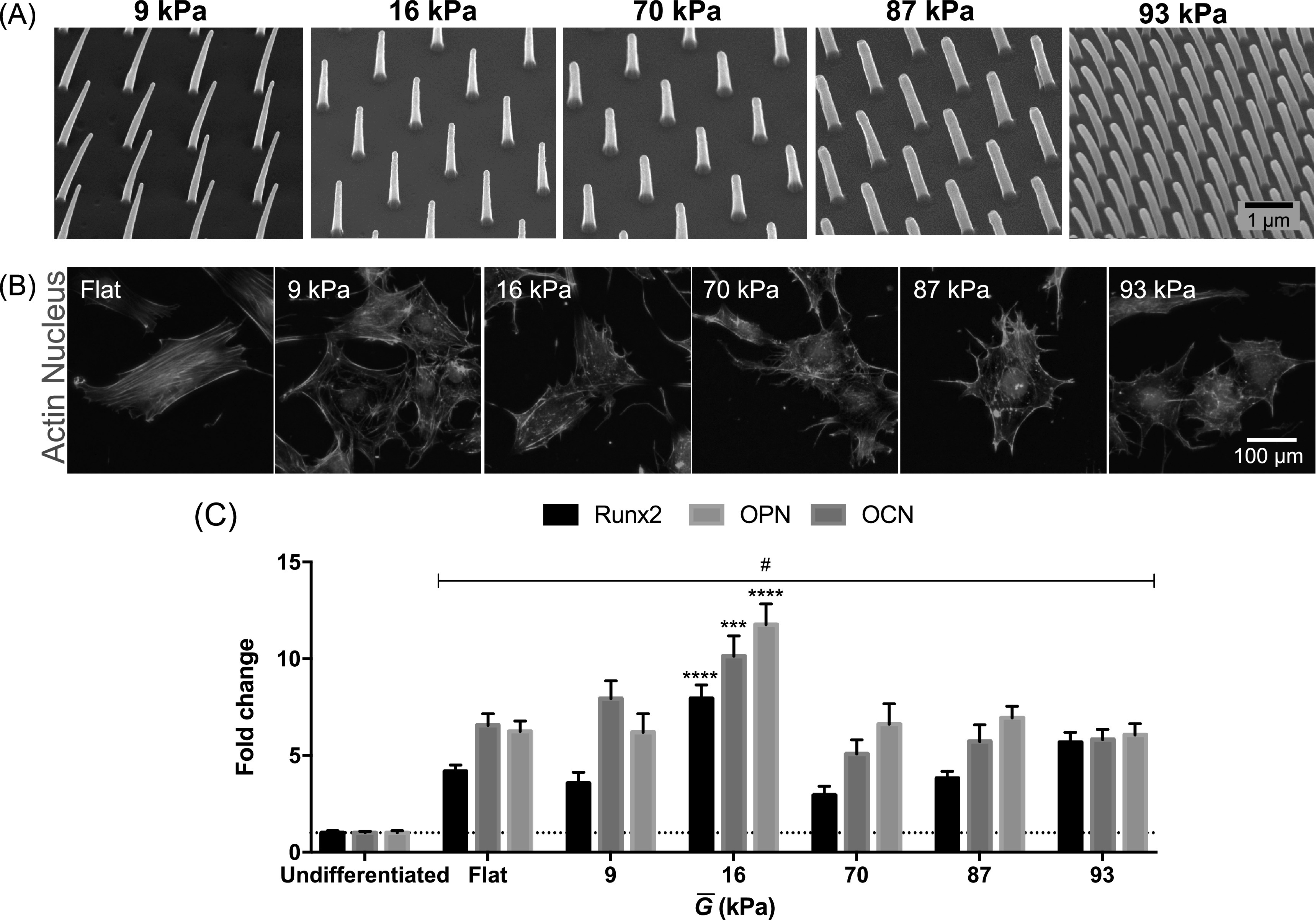
Substrate rigidity induces osteogenic differentiation. Substrate rigidity was controlled by varying the dimensions of high aspect ratio pillars. Substrate rigidity is reported as shear rigidity $\bar{G}.$ (A) SEM of high aspect ratio pillars with different $\bar{G}$ resulting from variations in pillar diameter and pitch. The pitch is kept at 1 *μ*m for all pillars except those that mimic shear rigidity of 87 kPa. (B) Representative images of MC3T3 pre-osteoblast cells grown on varying rigidities after 24 h. Cells were stained against actin (green) and nucleus (blue). Pre-osteoblast cells on varied substrate rigidities manifested drastic changes in morphology, especially size, filopodial formation and actin cytoskeleton organization compared to Flat. Quantification of osteoblast morphology is presented in figure S3. (C) Expression of osteogenic genes Runx family transcription factor 2 (*RUNX2)*, osteopontin (*OPN*) and osteocalcin (*OCN*) in MC3T3 pre-osteoblasts after 10 d of culture. # denotes statistically significant increase in osteogenic gene expression induced by all substrate rigidities for all genes compared to an Undifferentiated control (day 0). Fold change was calculated from *f* * denotes statistically significant increase in gene expression compared to Flat control. *** denotes *p* < 0.0005, **** denotes *p* < 0.0001. Statistical significance was measured using two-way ANOVA with Dunnett’s post hoc test. For comparison, Flat polycarbonate has shear modulus of 0.85 × 10^6^ kPa.

Osteogenic differentiation has been robustly shown to accelerate with higher substrate stiffness [36]. We fabricated a multiwell array with high aspect ratio nanopillars of varying diameter, pitch and height, to obtain surfaces that differ in stiffnesses (figure 4(A)). Altering substrate stiffness by changing nanopillar dimensions is a highly controllable way of altering the rigidity compared to e.g. hydrogel stiffness that relies on tweaking chemical concentration or UV-light exposure. Furthermore, this extends the range of substrate rigidities available and removes complications of coupled biochemical and biophysical properties arising from chemically-defined biomaterials such as polyacrylamide [53]. Cylindrical nanopillar arrays and bulk substrate mechanical properties have previously been demonstrated to be comparable using the effective shear modulus $\bar{G},$ calculated as follows [34]:

1 \begin{eqnarray*}\bar{G}=\displaystyle \frac{3}{16}{\left(\displaystyle \frac{d}{l}\right)}^{2}fE,\end{eqnarray*}

where *d* is the diameter of the pillar tip, *l* is the length, or height, of the nanopillar, *E* is the Young’s modulus of the bulk material, and *f* is the fill factor of the array.

As the pillars demonstrated here differ in morphology from an ideal cylinder, the deflection characteristics of the pillars had to be established, and the effective shear modulus amended to account for this, which we will call $\bar{G}^{\prime} .$ This was calculated using finite element analysis (see Methods for details), and the discrepancy between the ideal cylinder and the modelled pillar calculated by comparing their spring constants, ${\rm{\Delta }}k.$ As this value is a constant, the amendment is simple:

2 \begin{eqnarray*}\bar{G}^{\prime} =\bar{G}\,\ast \,{\rm{\Delta }}k.\end{eqnarray*}

For comparison to bulk substrates, the shear modulus and the Young’s modulus are related by the Poisson’s ratio, providing that the substrate is, or can be treated as, isotropic:

3 \begin{eqnarray*}G=\displaystyle \frac{E}{2\,\ast \,\left(1+\nu \right)},\end{eqnarray*}

where *ν* is Poisson’s ratio of the material. Dimensions of the high aspect ratio nanopillars and the corresponding mechanical properties are given in table S3.

We cultured MC3T3 pre-osteoblast cells on high aspect ratio nanopillars without addition of biochemical inducers of osteogenesis. After 24 h of culture, morphological differences in response to shear moduli were already manifested (figures 4(B) and S3). Osteoblasts showed decreasing actin texture (indicating less fibrillar actin structures) on all high aspect ratio pillars compared to Flat (with shear modulus of 0.85 × 10^6^ kPa). On Flat MC3T3 pre-osteoblasts showed spread morphology with organization of actin into highly aligned stress fibers highly characteristic of response on relatively stiff substrates. In contrast, cells on 9 and 16 kPa rigidities induced formation of cortical actin and showed cells with circular shapes. Additionally, osteoblasts on high aspect ratio nanopillars with rigidity of 9, 70 and 87 kPa showed statistically significant increase in the uniformity of actin radial distribution compared to Flat. We observed this particularly for substrates with 70 and 87 kPa rigidity, where cells showed long filopodial extensions but less circular and more elongated cell shapes compared to softer substrates. Increasing the rigidity to 93 kPa induced actin organization similar to substrates with 70 and 87 kPa rigidity, but reduced filopodial extensions.

After 10 d of culture, all substrate rigidities and Flat significantly increased expression of osteogenic genes Runx family transcription factor 2 (*RUNX2)*, osteopontin (*OPN*) and osteocalcin (*OCN*) compared to an Undifferentiated control (figure 4(C)). This was unsurprising as pre-osteoblasts tend to differentiate with increasing confluence [54]. Though potent in changing MC3T3 pre-osteoblast morphology, substrate rigidities of 9, 70, 87 and 93 kPa showed similar osteogenic profile to a Flat control. Only the high aspect ratio nanopillars with 16 kPa shear modulus significantly increased expression for all three osteogenic markers compared to Flat.

## Discussion and conclusion

Finding the right engineered substrate to influence cell behavior is key for new *in vitro* tools and models of *in vivo* behavior, and for potential regenerative purposes. Without the ability to quickly engineer arrays of patterns specifying topographies or rigidities, discovery of positive hits remains limited and inefficient. Here, we fabricated a multiwell array that allows for multiple topographical or mechanical conditions to be assessed simultaneously and in isolation. We showed that the multiwell array can be customized to contain a wide array of mechanical or topographical cues in a high-throughput fashion, allowing assessment of behavioral changes of various cell types within the same platform. Tested against different cell types and using a variety of analytical techniques, we exhibited the flexibility and capability of the multiwell arrays for screening engineered substrates. The advantages of the multiwell array over currently available screening platforms [26–28] are manifold, as discussed below.

First, the highly bespoke nature of the multiwell array enables creation of multitudes of mechanical and topographical cues to alter the cell microenvironment. Here, we have shown successful integration of a wide variety of patterns with different shapes (pillars versus grooves), length scales (nano- and micron-sized grooves), and effective rigidities (high aspect ratio pillars) in the multiwell array. Essentially, our method for multiwell array fabrication allows for any potential microenvironment exhibiting with geometric, topographical or mechanical properties to be mimicked with nanometer-scale precision. With our multiwell array method, both pattern replication throughput and fidelity, and cytocompatibility are improved. The current best patterned array available today utilizes the elastomer polydimethylsiloxane, which requires long curing times that ensure pattern replication but prevent high throughput production, may leak uncured oligomers toxic to cells [55], and provides a less reliable chemical interface [56].

The multiwell array indeed lends large flexibility in configuration, allowing the end-user to explicitly make arrays specifically optimized for the task at hand. Aside from full customization in the patterned cues, the multiwell array can be scaled up to a full-sized well plate and customized to larger well plate formats. With larger arrays for cell growth, the multiwell array can be converted from a screening to an *in vitro* cell culture device. For instance, multiwell arrays formatted with 12-wells and patterned with 127 nm tall nanopillars could be manufactured as a new cell culture tool for improved chondrocyte maintenance. Multiwell arrays with high aspect ratio nanopillars of 16 kPa shear rigidity could be used as an alternative and low-cost method to stimulate osteogenesis compared to use of recombinant growth factors.

To elevate the variety of cell signals presented in the multiwell array, chemical cues may also be coated onto a flat polystyrene slide then welded to the bottomless well plate [57, 58]. Other thermoplastics (e.g. polyurethane [35]) or metal/thermoplastic and ceramic/thermoplastic composites [59] amenable to forming complex microscale structures using injection moulding could be explored. The customizability of the mulitwell array truly permits screening of biophysical and biochemical environments.

Second, the multiwell array can be used for various analytical modes with standard laboratory equipment. One of the pitfalls of the currently available platforms is limitation of biological assessment to imaging techniques. Here, we showed that the multiwell array can be used with standard laboratory techniques such as fluorescent immunochemistry, qPCR, plate readers and microscopy. This allows for comprehensive examination of cell behavior induced by the engineered substrates on a genetic, morphological and functional level. We also exhibited that high-speed microscopic techniques for physiological measurements are possible using the multiwell array. Since containment is integrated with the patterned cues of interest, light only needs to with a single substrate. In terms of real-time techniques such as time lapse microscopy, the multiwell array also provides a stable platform that allows multiple locations to be assessed at once without regard to substrate drift. In contrast, standalone substrates (e.g. those made from soft lithography techniques) that require containment within a well will have two substrates interacting with light (i.e. the well plate and substrate on which cells adhere), and, depending on density, may be free floating in liquid. One limitation is the need for an injection moulding machine to rapidly produce bespoke multiwell arrays. While not all institutes have access to an injection moulding machine, new or established collaborations with institutes or biomedical industries (e.g. producers of conventional tissue culture plasticware) owning an injection moulding machine could easily overcome this. Production costs using an injection moulding machine is low, a negligible issue for individual laboratory groups wishing to customise and outsource production of multiwell arrays for their own scientific interest.

Third, the multiwell array format provides individual patterned areas in isolation. Studies through time have shown that cells rapidly change their paracrine environment (e.g. metabolites, cytokines and miRNAs) in response to subtle changes in the biochemical [60–62] and biophysical [63] milieu. This conflates signals that determine the true effect of a biophysical cue to cell behavior. Recently published screening platforms, like the biosurface structure array (BSSA) [28], the topographical chip (TopoChip) [26, 29], and the multiarchitectural chip (MARC) [27, 64] suffer from this issue because all biophysical cues exist together in the same container. Release of paracrine signals from cells influenced by one type of biophysical cue is extremely likely to influence behavior of cells on another type of cue. It is exactly this conflation of paracrine signals that preclude population-based assays such as qPCR from being utilized on these screening platforms. By separating individual substrate stiffnesses or topographical cues, as done in the multiwell array, crosstalk between substrates is avoided and biological results are therefore inferred to arise only from one substrate type.

Taken together, the multiwell array uniquely combines high-throughput production, flexibility in topographical and rigidity cues, and quality and customizability that no other screening platform to date offers. Currently, no array format exists that allows for screening of cell response to a range of tailored biophysical properties of the substrate simultaneously and independently.

## Materials and methods

### Nanopillars master stamp fabrication

A quartz substrate coated with a bilayer of poly(methyl methacrylate) (PMMA) was written with dots using EBL (Vistec VB6) using an ‘on the fly’ strategy as previously described [65] After development in 1:1 methyl isobutyl ketone:isopropyl alcohol (MIBK:IPA) at 23 °C, a 50 nm nichrome (NiCr) film was evaporated and lift-off performed in 50 °C acetone for 12 h. A sequence of five masked etches were then performed, alternating exposed patterns at each step and varying the etch depth. A positive-tone photoresist (Shipley S1818, microposit) was spun at 3000 rpm, exposed for 4.5 s on a mask aligner (Suss MA6), and developed in 1:1 microposit developer:water for 75 s. Nanopillar patterns were etched into the quartz substrate in a trifluoromethane/argon (CHF_3_/Ar) plasma in a reactive ion etching (RIE, Oxford RIE 80+) tool. Photoresist was removed in acetone, and the process was repeated with a different mask configuration until the 20 nanopillar patterns were etched to 20 different heights (5 iterations total). The slide was coated with a fluorosilane anti-stick layer, and an SU-8 epoxy photoresist/Cirlex polyimide (DuPont) hybrid inlay for injection moulding was patterned as a negative relief of the master using NIL as described previously [38].

### Grooves master stamp fabrication

A silicon wafer was coated with a 200 nm film of a positive-tone resist (CSAR 62, AllResist) and patterns exposed using EBL with exposure time approximately 7 h for 6.3 cm^2^. Patterns were arranged in an 8 by 3 array on 9 mm center-to-center pitch. Blank control regions were also included, and the pattern locations were randomized in the array. After EBL exposure, the wafer was developed in *n*-amyl acetate at 23 °C and rinsed thoroughly in IPA. Grooves were transferred into the silicon substrate using sulfur hexafluoride/octafluorocyclobutane (SF_6_/C_4_F_8_) etching (STS inductively coupled plasma) to a depth of 250 nm. The remaining positive resist was removed in acetone. A NIL machine (EVG 5200) was used to create a polymer replica (SmartNIL foil) as a working stamp that was cut to size, mounted and used for injection moulding.

High aspect ratio nanopillars master stamp fabrication: A quartz slide was spincoated with a bilayer of PMMA, and the pattern was written using EBL. The pattern was developed for 1 min in 2.5:1 MIBK:IPA solution, and rinsed with IPA for 30 s. Residual PMMA in the nanopits was removed using a 30 s 80 W O_2_ plasma treatment, and an 80 mm thick layer of nickel was thereafter deposited. This was removed using the N-Methyl-2-pyrrolidone remove (remover 1165, microposit) at 50 °C for 12 h to form nanodots on nickel. These were then etched into nanopillar arrays using a CHF_3_/Ar plasma using reactive ion etching (Oxford RIE 80+) for 33 min in a single etch step process. A polymer replica was then created from the nanopillar arrays through NIL of the SU-8/Cirlex hybrid inlay (similar to the process of fabricating the nanopillars array). The SU-8/Cirlex replica was finally used for injection moulding.

### Injection moulding

Nanopillars, grooves and high aspect ratio pillars were injection moulded using polymer replica inserts mounted in a custom tooling configuration in an Engel Victory 28 injection moulding machine [38, 66]. The resulting polymer slides contained replica of structures found in the original master stamp (on either silicon or quartz) or negative relief of the polymer replica (on either smartNIL foil, nickel or SU-8/Cirlex polyimide). Nanopillars and grooves were injection mouleded in polystyrene (1810 crystal polystyrene, Total, Belgium), as previously described [35, 38]. The bottomless plate with individual well dimensions matching that of a standard 96 well plate (0.3 cm^2^ culture area) were injection moulded in polystyrene and made in house.

Polystyrene was unsuitable for injection moulding of high aspect ratio nanopillars due to its relatively low glass transition temperature that results in degradation of pillar shapes and mechanical properties. Due to stretching in during injection moulding, use of polycarbonate leads to high aspect ratio nanopillars with features taller and thinner than the quartz master counterparts [67], therefore injection moulding of these pillars was carried out using Markrolon^®^ OD2015 Polycarbonate.

Currently, there are no tools that allow accurate empirical measurement of the deformation and rigidity of high aspect ratio pillars with nanometer length scale. To overcome this limitation and ensure accurate approximation of the rigidity of high aspect ratio pillars, we used finite element analysis. Finite element analysis has been used in determining rigidity of sub-millimetre pillar arrays, which operates under the same structural mechanical principles of metre-scaled cantilever beams [68]. Using the same principle, we categorised the mechanical properties of the resulting high aspect ratio nanopillars using finite element modelling (COMSOL Multiphysics). Typical Euler–Bernoulli constraints for cantilever beams were used: (1) every part of the pillar is free to move except from the base, which is fixed (and extremely rigid compared to the pillar tops); and (2) that the load exerted by the cells on each pillar is a horizontal point load at the top of the pillars. The changing cross-sectional areas of our high aspect ratio pillars were also taken into account. From finite element modelling we obtained the spring constant of high aspect ratio pillars, allowing us to extrapolate effective shear and Young’s moduli, using the equations (1)–(3) described above.

### Preparation of substrates for cell seeding

All slide arrays were attached to the bottomless multiwell plate by ultrasonic welding (Standard 2000, Rinco Ultrasonics) to create the final multiwell array. Prior to cell seeding, multiwell arrays nanopillars and grooves were cleaned with 70% ethanol and distilled deionized water. then UV sterilized. High aspect ratio pillars were lightly cleaned using compressed air to prevent collapse of nanopillars. All substrates were thereafter treated with O_2_ plasma (80 W, 1 min) then UV sterilized for 20 min prior to cell seeding.

### HiPSC-CM cell culture and functionality assays

hiPSC-CM (NCardia) were cultured following the manufacturer’s protocol and proprietary media at a cell density of 100 000 cells cm^−2^. Prior to plating, multiwell groove array was coated with human fibronectin (10 *μ*g ml^−1^, R&D Systems) for 1 h, then washed twice with phosphate buffered saline (PBS, Sigma-Aldrich). On day 10, cells were loaded with the voltage sensitive fluorescent dye FluoVolt (1:1000, ThermoFisher) along with Powerload (1:100, ThermoFisher) in serum-free medium.and incubated for 25 min at 37 °C. Subsequently, action potentials were recorded using the CellOPTIQ^®^ system (Clyde Biosciences) at 10 000 fps as the depolarization time of cardiomyocytes is between 5 and 10 ms. Additionally, contractility analysis was done by recording videos at 100 fps that were analyzed using the MuscleMotion software [69].

### Chondrocyte cell culture

Isolation of costal chondrocytes were performed as described [70]. After isolation, murine chondrocytes were cultured in alpha minimum essential medium supplemented with ascorbic acid, glutamate, sodium pyruvate, 10% fetal bovine serum (FBS) and 1% penicillin/streptomycin. Extracted chondrocytes were either used after routine culture in standard tissue culture plastic (cultured chondrocytes) or immediately after harvest (isolated chondrocytes). Chondrocytes were seeded on nanopillars at 2500 cells cm^−2^ in 100 *μ*l complete media, with medium change every 2 d. Chondrocytes were tested for viability, harvested for gene expression analysis at specific timepoints, or fixed for immunohistochemistry and immunofluorescence, as described below.

### MC3T3 cell culture

MC3T3 pre-osteoblasts (ATCC) were cultured using minimum essential medium alpha without ascorbic acid and containing 10% FBS and 1% penicillin/streptomycin for 10 d. MC3T3 were seeded on high aspect ratio nanopillars at 5000 cells cm^−2^ and 100 *μ*l complete media. MC3T3 were harvested for immunofluorescence staining at 24 h after culture and gene expression analysis after 10 d of culture.

### Immunofluorescence staining and imaging

At selected timepoints, cells were fixed with 4% paraformaldehyde and permeabilized with 0.1% Triton-X 100. Then, samples were blocked with 1% bovine serum albumin and 10% goat serum in PBS. hiPSC-CM were stained against α-actinin (E7732, Sigma, 1:500) using an Alexa Fluor 488-conjugated goat-anti-mouse-antibody (Life Technologies, 1:500) secondary. Chondrocytes and MC3T3 were stained against actin using Alexa Fluor 488-phalloidin (LifeTechnologies). NucBlue fixed cell stain (LifeTechnologies) was used to stain the nuclei of the cells. Imaging was performed under a 10× (numerical aperture 0.3), 20× (numerical aperture 0.45) or 40× magnification (numerical aperture 0.6) using an EVOS FL2 Auto microscope, (ThermoFisher). Both chondrocyte and MC3T3 morphology changed across different biophysical stimuli were measured using image-based cell profiling, as previously described [51, 71–75].

### RNA harvest and qPCR

At specified timepoints, total RNA was harvested from cells (ReliaPrep Cell RNA extraction kit, Promega). Relative gene expression was measured from a total of 5 ng RNA using a one-step qPCR kit with SYBR dye (PrimerDesign) and normalized to GAPDH or 18 S ribosomal RNA housekeeping gene. A list of the forward and reverse primers used are given in table S4.

### Proliferation rate analysis

Metabolic rate was used as a surrogate marker for chondrocyte proliferation. At selected time points, chondrocytes on nanopillar arrays were added with PrestoBlue reagent (ThermoFisher Scientific, 1:100 dilution). Fluorescence of the reduced reagent was measured at 590 nm emission and 560 nm excitation using a microplate reader (Tecan Infiniti Pro) and was normalized to cultured chondrocytes on Flat at day 7.

### Alcian blue staining and quantification

At different time points, cultured chondrocytes grown on nanopillar arrays were fixed with 4% paraformaldehyde for 15 min at 4 °C. Thereafter, each well was incubated with 0.1% Alcian blue 8GX (Sigma Aldrich) dissolved in 0.1 N hydrochloric acid in phosphate buffered saline for 30 min. Subsequently, a flatbed scanner was used to take color images (at 1200 pixels per image) of the nanopillar arrays. White balance correction of nanopillar array image was performed before image deconvolution to extract the Alcian blue stain. Measurement of Alcian blue intensity was performed using the color deconvolution plugin for ImageJ (National Institutes of Health). All intensity measurements were normalized to those on Flat at day 7.

### Statistical analysis

All data are presented as mean ± standard deviation. Statistical analysis was performed using GraphPad Prism v7.0. One-way ANOVA with Tukey’s post hoc test or two-way ANOVA with Dunnett’s post hoc test was used, with *p* < 0.05 considered significant.

## Author contributions

NG conceptualized the study. EH and MFAC carried out biological studies on multiwell arrays. FAC, AS, RL and PMR made multiwell arrays. EH, MFAC, FAC, and NG wrote and edited the manuscript with contributions from other authors. All authors have given approval to the final version of the manuscript.

## Funding sources

European research council FAKIR 648892 Consolidator Award

British Heart Foundation 4-year PhD program

Engineering and Physical Sciences Research Council (EPSRC) PhD funding—ref 1651390

Biotechnology and Biological Sciences Research Council (BBSRC) BB/K011235/1

## Abbreviations

ACAN, aggrecan; APD50, action potential duration at 50% of the amplitude; CD50, contraction duration at 50% of the amplitude; CHF_3_/Ar, trifluoromethane/argon; COL1A, collagen type 1a; COL2A, collagen type 2a; CoV, coefficient of variation; DMEM, dulbecco’s modified Eagle’s medium; EBL, electron beam lithography; fps, frames per second; IPA, isopropyl alcohol; MIBK, methyl isobutyl ketone; NiCr, nichrome; NIL, nanoimprint lithography; NMP, N-Methyl-2-pyrrolidone; OCN, osteocalcin; OPN, osteopontin; qPCR, quantitative polymerase chain reaction; RIE, reactive ion etching; RUNX2, Runx family transcription factor 2; SD, standard deviation; SEM, scanning electron microscope; SF_6_/C_4_F_8_, sulfur hexafluoride/octafluorocyclobutane; SOX9, SRY-box 9; T_Contraction_, contraction time; T_Relaxation_, relaxation time.

## References

[1] Junttila M R, De Sauvage F J (2013). Influence of tumour micro-environment heterogeneity on
therapeutic response. Nature.

[2] Lu P, Weaver V M, Werb Z (2012). The extracellular matrix: a dynamic niche in cancer
progression. J. Cell Biol..

[3] Frangogiannis N G (2017). The extracellular matrix in myocardial injury, repair, and
remodeling. J. Clin. Invest..

[4] Walker C, Mojares E, Del Río Hernández A (2018). Role of extracellular matrix in development and cancer
progression. IJMS.

[5] Bettinger C J, Langer R, Borenstein J T (2009). Engineering substrate topography at the micro- and nanoscale to
control cell function. Angew. Chem. Int. Ed..

[6] Rizwan M, Peh G S, Adnan K, Naso S L, Mendez A R, Mehta J S, Yim E K F (2016). *In Vitro* topographical model of fuchs dystrophy
for evaluation of corneal endothelial cell monolayer
formation. Adv. Healthcare Mater..

[7] Cutiongco M F A, Goh S-H, Aid-Launais R, Le Visage C, Yee L H, Yim E K F (2016). Planar and tubular patterning of micro and nano-topographies on
poly(Vinyl Alcohol) hydrogel for improved endothelial cell
responses. Biomaterials.

[8] Guilak F, Cohen D M, Estes B T, Gimble J M, Liedtke W, Chen C S (2009). Control of stem cell fate by physical interactions with the
extracellular matrix. Cell Stem Cell.

[9] Bressel T A B, de Queiroz J D F, Gomes Moreira S M, Da Fonseca J T, Filho E A, Guastaldi A C, Batistuzzo de Medeiros S R (2017). Laser-modified titanium surfaces enhance the osteogenic
differentiation of human mesenchymal stem cells. Stem Cell Res. Ther..

[10] Kim J H, Park B G, Kim S K, Lee D H, Lee G G, Kim D H, Choi B O, Lee K B (2018). Nanotopographical regulation of pancreatic islet-like cluster
formation from human pluripotent stem cells using a gradient-pattern
chip. Acta Biomater..

[11] Bucaro M A, Vasquez Y, Hatton B D, Aizenberg J (2012). Fine-tuning the degree of stem cell polarization and alignment on
ordered arrays of high-aspect-ratio nanopillars. ACS Nano.

[12] Sala A, Hänseler P, Ranga A, Lutolf M P, Vörös J, Ehrbar M, Weber F E (2011). Engineering 3D cell instructive microenvironments by rational
assembly of artificial extracellular matrices and cell
patterning. Integr. Biol..

[13] Schmidt C E, Baier J M (2000). Acellular vascular tissues: natural biomaterials for tissue
repair and tissue engineering. Biomaterials.

[14] Yang K, Jung H, Lee H-R, Lee J S, Kim S R, Song K Y, Cheong E, Bang J, Im S G, Cho S-W (2014). Multiscale, hierarchically patterned topography for directing
human neural stem cells into functional neurons. ACS Nano.

[15] Luo T, Mohan K, Iglesias P A, Robinson D N (2013). Molecular mechanisms of cellular mechanosensing. Nat. Mater..

[16] Geiger B, Spatz J P, Bershadsky A D (2009). Environmental sensing through focal adhesions. Nat. Rev. Mol. Cell Biol..

[17] Vogel V, Sheetz M (2006). Local force and geometry sensing regulate cell
functions. Nat. Rev. Mol. Cell Biol..

[18] Huang C, Holfeld J, Schaden W, Orgill D, Ogawa R (2013). Mechanotherapy: revisiting physical therapy and recruiting
mechanobiology for a new era in medicine. Trends Mol. Med..

[19] Yim E K F, Pang S W, Leong K W (2007). Synthetic nanostructures inducing differentiation of human
mesenchymal stem cells into neuronal lineage. Exp. Cell. Res..

[20] Dalby M J, Gadegaard N, Tare R, Andar A, Riehle M O, Herzyk P, Wilkinson C D W, Oreffo R O C (2007). The control of human mesenchymal cell differentiation using
nanoscale symmetry and disorder. Nat. Mater..

[21] Chan L Y, Birch W R, Yim E K F, Choo A B H (2013). Temporal application of topography to increase the rate of neural
differentiation from human pluripotent stem cells. Biomaterials.

[22] Cutiongco M F A, Chua B M X, Neo D J H, Rizwan M, Yim E K F (2018). Functional differences between healthy and diabetic endothelial
cells on topographical cues. Biomaterials.

[23] Dickinson L E, Rand D R, Tsao J, Eberle W, Gerecht S (2012). Endothelial cell responses to micropillar substrates of varying
dimensions and stiffness. J. Biomed. Mater. Res. A.

[24] Liliensiek S J, Wood J A, Yong J, Auerbach R, Nealey P F, Murphy C J (2010). Modulation of human vascular endothelial cell behaviors by
nanotopographic cues. Biomaterials.

[25] Tan K K B, Tann J Y, Sathe S R, Goh S-H, Ma D, Goh E L K, Yim E K F (2015). Enhanced differentiation of neural progenitor cells into neurons
of the mesencephalic dopaminergic subtype on topographical
patterns. Biomaterials.

[26] Unadkat H V (2011). An algorithm-based topographical biomaterials library to instruct
cell fate. Proc. Natl Acad. Sci. USA.

[27] Moe A A K (2012). Microarray with micro- and nano-topographies enables
identification of the optimal topography for directing the differentiation
of primary murine neural progenitor cells. Small.

[28] Markert L D (2009). Identification of distinct topographical surface microstructures
favoring either undifferentiated expansion or differentiation of murine
embryonic stem cells. Stem Cells Dev..

[29] Hulshof F F B, Zhao Y, Vasilevich A, Beijer N R M, de Boer M, Papenburg B J, van Blitterswijk C, Stamatialis D, De Boer J (2017). Nanotopochip: high-throughput nanotopographical cell
instruction. Acta Biomater..

[30] Su N, Gao P L, Wang K, Wang J Y, Zhong Y, Luo Y (2017). Fibrous scaffolds potentiate the paracrine function of
mesenchymal stem cells: a new dimension in cell-material
interaction. Biomaterials.

[31] Valles G, Bensiamar F, Crespo L, Arruebo M, Vilaboa N, Saldana L (2015). Topographical cues regulate the crosstalk between mscs and
macrophages. Biomaterials.

[32] Shin H, Lee M N, Choung J S, Kim S, Choi B H, Noh M, Shin J H (2016). Focal adhesion assembly induces phenotypic changes and
dedifferentiation in chondrocytes. J. Cell. Physiol..

[33] Lee H-P, Gu L, Mooney D J, Levenston M E, Chaudhuri O (2017). Mechanical confinement regulates cartilage matrix formation by
chondrocytes. Nat. Mater..

[34] Rasmussen C H, Reynolds P M, Petersen D R, Hansson M, McMeeking R M, Dufva M, Gadegaard N (2016). Enhanced differentiation of human embryonic stem cells toward
definitive endoderm on ultrahigh aspect ratio nanopillars. Adv. Funct. Mater..

[35] Stormonth-Darling J M, Saeed A, Reynolds P M, Gadegaard N (2016). Injection molding micro- and nanostructures in thermoplastic
elastomers. Macromol. Mater. Eng..

[36] Engler A J, Sen S, Sweeney H L, Discher D E (2006). Matrix elasticity directs stem cell lineage
specification. Cell.

[37] Li B, Moshfegh C, Lin Z, Albuschies J, Vogel V (2013). Mesenchymal stem cells exploit extracellular matrix as
mechanotransducer. Sci. Rep..

[38] Stormonth-Darling J M, Gadegaard N (2012). Injection moulding difficult nanopatterns with hybrid polymer
inlays. Macromol. Mater. Eng..

[39] Zhang Z, Guan N, Li T, Mais D E, Wang M (2012). Quality control of cell-based high-throughput drug
screening. Acta Pharm. Sin. B.

[40] Huethorst E, Hortigon M, Zamora-Rodriguez V, Reynolds P M, Burton F, Smith G, Gadegaard N (2016). Enhanced human-induced pluripotent stem cell derived
cardiomyocyte maturation using a dual microgradient
substrate. ACS Biomater. Sci. Eng..

[41] McDevitt T C, Angello J C, Whitney M L, Reinecke H, Hauschka S D, Murry C E, Stayton P S (2002). *In Vitro* generation of differentiated cardiac
myofibers on micropatterned laminin surfaces. J. Biomed. Mater. Res..

[42] Bray M-A, Sheehy S P, Parker K K (2008). Sarcomere alignment is regulated by myocyte shape. Cell Motil. Cytoskeleton.

[43] Lundy S D, Zhu W-Z, Regnier M, Laflamme M A (2013). Structural and functional maturation of cardiomyocytes derived
from human pluripotent stem cells. Stem Cells Dev..

[44] Vigilante A (2019). Identifying extrinsic versus intrinsic drivers of variation in
cell behavior in human ipsc lines from healthy donors. Cell Rep..

[45] Schwartzentruber J (2018). Molecular and functional variation in iPSC-derived sensory
neurons. Nat. Genet..

[46] Huo J, Kamalakar A, Yang X, Word B, Stockbridge N, Lyn-Cook B, Pang L (2017). Evaluation of batch variations in induced pluripotent stem
cell-derived human cardiomyocytes from 2 major suppliers. Toxicol. Sci..

[47] Vitale A M, Matigian N A, Ravishankar S, Bellette B, Wood S A, Wolvetang E J, Mackay-Sim A (2012). Variability in the generation of induced pluripotent stem cells:
importance for disease modeling. Stem Cells Transl. Med..

[48] Bot C T, Juhasz K, Haeusermann F, Polonchuk L, Traebert M, Stoelzle-Feix S (2018). Cross-site comparison of excitation-contraction coupling using
impedance and field potential recordings in hiPSC
cardiomyocytes. J. Pharmacol. Toxicol. Methods.

[49] Blinova K (2018). International multisite study of human-induced pluripotent stem
cell-derived cardiomyocytes for drug proarrhythmic potential
assessment. Cell Rep..

[50] Solchaga L A, Penick K, Goldberg V M, Caplan A I, Welter J F (2010). Fibroblast growth factor-2 enhances proliferation and delays loss
of chondrogenic potential in human adult bone-marrow-derived mesenchymal
stem cells. Tissue Eng. A.

[51] Cutiongco M F, Jensen B S, Reynolds P M, Gadegaard N (2018). Predicting gene expression using morphological cell responses to
nanotopography.

[52] Jalal S, Shi S, Acharya V, Huang R Y-J, Viasnoff V, Bershadsky A D, Tee Y H (2019). Actin cytoskeleton self-organization in single epithelial cells
and fibroblasts under isotropic confinement. J. Cell Sci..

[53] Trappmann B (2012). Extracellular-matrix tethering regulates stem-cell
fate. Nat. Mater..

[54] Yan X-Z, Yang W, Yang F, Kersten-Niessen M, Jansen J A, Both S K (2014). Effects of continuous passaging on mineralization of MC3T3-E1
cells with improved osteogenic culture protocol. Tissue Eng. C.

[55] Regehr K J, Domenech M, Koepsel J T, Carver K C, Ellison-Zelski S J, Murphy W L, Schuler L A, Alarid E T, Beebe D J (2009). Biological implications of polydimethylsiloxane-based
microfluidic cell culture. Lab Chip.

[56] Bodas D, Khan Malek C (2006). Formation of more stable hydrophilic surfaces of pdms by plasma
and chemical treatments. Microelectron. Eng..

[57] Alexander M R, Wildman R D, Begines B, Hook A L, Tuck C J (2016). Development, printability and post-curing studies of formulations
of materials resistant to microbial attachment for use in inkjet based 3D
printing. Rapid Prototyping J..

[58] Pang S, Sun M, Huang Z, He Y, Luo X, Guo Z, Li H (2019). Bioadaptive nanorod array topography of hydroxyapatite and
TiO. J. Biomed. Mater. Res. A.

[59] Piotter V, Benzler T, Gietzelt T, Ruprecht R, Haußelt J (2000). Micro powder injection molding. Adv. Eng. Mater..

[60] Narayanan R, Huang C C, Ravindran S (2016). Hijacking the cellular mail: exosome mediated differentiation of
mesenchymal stem cells. Stem Cells Int..

[61] Laurenzana I (2018). Extracellular vesicles: a new prospective in crosstalk between
microenvironment and stem cells in hematological
malignancies. Stem Cells Int..

[62] Guescini M, Maggio S, Ceccaroli P, Battistelli M, Annibalini G, Piccoli G, Sestili P, Stocchi V (2017). Extracellular vesicles released by oxidatively injured or intact
c2c12 myotubes promote distinct responses converging toward
myogenesis. IJMS.

[63] Tsimbouri P M, McMurray R J, Burgess K V, Alakpa E V, Reynolds P M, Murawski K, Kingham E, Oreffo R O C, Gadegaard N, Dalby M J (2012). Using nanotopography and metabolomics to identify biochemical
effectors of multipotency. ACS Nano.

[64] Ankam S, Suryana M, Chan L Y, Moe A A K, Teo B K K, Law J B K, Sheetz M P, Yee L H, Yim E K F (2013). Substrate topography and size determine the fate of human
embryonic stem cells to neuronal or glial lineage. Acta Biomater..

[65] Gadegaard N, Thoms S, Macintyre D S, Mcghee K, Gallagher J, Casey B, Wilkinson C D W (2003). Arrays of nano-dots for cellular engineering. Microelectron. Eng..

[66] Reynolds P M, Pedersen R H, Stormonth-Darling J, Dalby M J, Riehle M O, Gadegaard N (2013). Label-free segmentation of Co-cultured cells on a
nanotopographical gradient. Nano Lett..

[67] Stormonth-Darling J M, Pedersen R H, How C, Gadegaard N (2014). Injection moulding of ultra high aspect ratio nanostructures
using coated polymer tooling. J. Micromech. Microeng..

[68] Fu J, Wang Y-K, Yang M T, Desai R A, Yu X, Liu Z, Chen C S (2010). Mechanical regulation of cell function with geometrically
modulated elastomeric substrates. Nat. Methods.

[69] Sala L (2018). MUSCLEMOTION: a versatile open software tool to quantify
cardiomyocyte and cardiac muscle contraction *in vitro* and
*in vivo*. Circ. Res..

[70] Gosset M, Berenbaum F, Thirion S, Jacques C (2008). Primary culture and phenotyping of murine
chondrocytes. Nat. Protocols.

[71] Caicedo J C (2017). Data-analysis strategies for image-based cell
profiling. Nat. Methods.

[72] McQuin C (2018). Cell profiler 3.0: next-generation image processing for
biology. PLoS Biol..

[73] Jones T R (2009). Scoring diverse cellular morphologies in image-based screens with
iterative feedback and machine learning. Proc. Natl Acad. Sci..

[74] Kong H J, Polte T R, Alsberg E, Mooney D J (2005). FRET measurements of cell-traction forces and nano-scale
clustering of adhesion ligands varied by substrate stiffness. Proc. Natl Acad. Sci..

[75] Rostam H M, Reynolds P M, Alexander M R, Gadegaard N, Ghaemmaghami A M (2017). Image based machine learning for identification of macrophage
subsets. Sci. Rep..

